# Transcranial direct current stimulation for chronic headaches, a randomized, controlled trial

**DOI:** 10.3389/fpain.2024.1353987

**Published:** 2024-02-27

**Authors:** Jill Angela Hervik, Karl Solbue Vika, Trine Stub

**Affiliations:** ^1^ Department of Anaesthesiology, Vestfold Hospital Trust, Tonsberg, Norway; ^2^ Department of School and Nursery, NIFU Nordic Institute for Studies in Innovation, Research and Education, Oslo, Norway; ^3^ Department of Community Medicine, National Research Center in Complementary and Alternative Medicine, NAFKAM, UiT The Arctic University of Norway, Tromsø, Norway

**Keywords:** chronic headache, neuromodulation, transcranial direct current stimulation, tDCS, daily function, pain, randomized controlled trial

## Abstract

**Background and objectives:**

Chronic headaches are a frequent cause of pain and disability. The purpose of this randomized trial was to examine whether transcranial direct current stimulation (tDCS) applied to the primary motor cortex, reduces pain and increases daily function in individuals suffering from primary chronic headache.

**Materials and methods:**

A prospective, randomized, controlled trial, where participants and assessors were blinded, investigated the effect of active tDCS vs. sham tDCS in chronic headache sufferers. Forty subjects between 18 and 70 years of age, with a diagnosis of primary chronic headache were randomized to either active tDCS or sham tDCS treatment groups. All patients received eight treatments over four consecutive weeks. Anodal stimulation (2 mA) directed at the primary motor cortex (M1), was applied for 30 min in the active tDCS group. Participants in the sham tDCS group received 30 s of M1 stimulation at the start and end of the 30-minute procedure; for the remaining 29 min, they did not receive any stimulation. Outcome measures based on data collected at baseline, after eight treatments and three months later included changes in daily function, pain levels, and medication.

**Results:**

Significant improvements in both daily function and pain levels were observed in participants treated with active tDCS, compared to sham tDCS. Effects lasted up to 12 weeks post-treatment. Medication use remained unchanged in both groups throughout the trial with no serious adverse effects reported.

**Conclusion:**

These results suggest that tDCS has the potential to improve daily function and reduce pain in patients suffering from chronic headaches. Larger randomized, controlled trials are needed to confirm these findings.

**Trial registration:**

The study was approved by the local ethics committee (2018/2514) and by the Norwegian Centre for Research Data (54483).

## 1 Background

Headache is a common complaint. Chronic headaches and intractable migraines can lead to a serious reduction in function and quality of life (1), imposing significant economic burdens on individuals, healthcare systems, and society (2). Tension-type headache has been declared the second most prevalent condition in the world (22%) by the Global Burden of Disease studies (3). Interestingly, headache prevalence, including migraine and non-migrainous headaches, remained stable, when trends were examined in Northern Norway during 2006–2008 (*n* = 51 836) and 2017–2019 (*n* = 39,561) (4). Female sex, chronic musculoskeletal complaints, and a high depression or anxiety score at baseline doubled the risk of headache incidence in these surveys. Sex disparity in chronic headache prevalence has been recognized for decades. Indeed, a review of headache prevalence studies published until 2020 (*n* = 357) revealed a female dominated gender ratio for chronic headaches. A global study indicated that active headache disorders of any type were present in 52.0% of the populations studied (males 44.4%, females 57.8%), migraine in 14.0% (males 8.6%, females 17.0%) and tension-type headache in 26.0% (males 23.4%, females 27.1%) (5).

In addition to tension-type headache, other non-migrainous chronic headaches include post-traumatic headaches, daily persistent headaches, and hemicrania continua. The International Headache Society defines chronic headache as a headache that occurs for at least 15 days or more a month, for longer than three months (6, 7). Primary headache disorders have no apparent underlying cause; symptoms include recurrent or persistent head pain (8).

A high degree of disability is associated with chronic headaches, often provoking negative changes in family life, social situations, and employment (9). Depression, anxiety, poor sleep and stress are often associated with headaches. There is evidence that these symptoms are potential prognostic factors for unfavorable preventative treatment outcomes (10). Diagnosis and treatment difficulties are present when tension-type headaches and migraine overlap and when individual episodes of pain start to merge (4).

Even though the International Headache Society [IHS] has introduced guidelines for the organization of headache service and management, there is no single standard of care for those with primary chronic headache symptoms (6). Patients often initiate treatment themselves without medical advice (11). The Norwegian Pharmaceutical Product Compendium recommends the use of weak painkillers such as Paracetamol, aspirin, or non-steroidal anti-inflammatory drugs [NSAID's] for tension-type headaches. However, long-term use can lead to gastrointestinal bleeding (12). Furthermore, codeine-based medicines can provoke headaches and are thus not recommended. The compendium suggests that antidepressants such as amitriptyline, mirtazapine, and venlafaxine may have an effect. Comorbidities such as cardiovascular disease restrict the use of these medicines and adverse effects include sleep problems, dry mouth, constipation, and weight gain. For patients who suffer from migraine, preventative medication typically includes calcitonin gene-related peptide [CGRP] inhibitors and triptans during attacks. Triptans bind to serotonin receptors in the brain diminishing vascular swelling. Serotonin toxicity is a risk when triptans and selective serotonin reuptake inhibitors [SSRIs] are simultaneously prescribed (15).

The International Association of Pain [IASP] (13) and the National Institute of Health and Care Excellence [NICE] (14) have recommended non-pharmacological approaches as first-line therapy for chronic pain, including chronic headache***.*** Neurostimulation is a suitable option for patients who do not want medication, in cases where adverse effects are problematic, or when first-line therapies are limited or have failed. Non-invasive treatments include vagus nerve stimulation, transcutaneous electrical nerve stimulation [TENS], repetitive transcranial magnetic stimulation [TMS] and transcranial direct current stimulation [tDCS] (16).

### 1.1 tDCS—mechanisms of action

tDCS occurs via a constant electric current produced by a battery-operated current generator. A weak current of 2 mA is delivered through two electrodes fixed to the scalp. tDCS exerts its effects through the modulation of the resting membrane potential of neural fibers. Modulation depends on the stimulation polarity where anodal or cathodal stimulation leads to depolarization or hyperpolarization respectively (17). Polarization direction depends on axonal orientations within the electric field (18). Effects are concentrated under the electrodes; though polarity, electrode size and placement precision, including the duration of stimulation are variables that can potentially influence distant neural networks (19). tDCS studies exploring chronic pain conditions during the last 30 years have mainly targeted the motor cortex [M1] (20). Studies investigating focal or lateralized headaches have mostly targeted the M1 on the hemisphere contralateral to the site of pain. Further, studies focusing on diffuse or non-lateralized pain have targeted the M1 on the left (dominant) hemisphere. Tension-type headache, the most prevalent type of chronic primary headache (6), usually affects the whole head. Consequently, anodal stimulation of the left M1 was considered appropriate in our study.

Functional magnetic resonance imaging [fMRI] studies have shown that noninvasive brain stimulation of the M1 modulates motor cortical function (21) and can enhance cortical plasticity in healthy humans, particularly in association with motor training (22). Stimulation of the M1 has shown increased excitability by reducing neuronal membrane potential to a greater degree, compared to stimulation of the sensory cortex [S1] and the dorsolateral prefrontal cortex [DLPFC] (23). Alterations in cortical excitability lasting beyond the time of stimulation have been related to decreased Gamma-aminobutyric acid [GABA], and longer-term potentiation of glutamate pathways involving *N*-methyl-D-aspartate [NMDA] receptors, specifically after stimulation of the left M1 (24). Although studies have demonstrated that the S1 and the DLPFC are involved in pain modulation (25), there is evidence for co-activation of the M1, S1, and DLPFC during pain processing (26), suggesting that stimulation of M1 by association can affect pain levels. Repeated cortical stimulation to induce more lasting and effective modulation has shown promising results in terms of efficacy and safety for the treatment of different chronic primary pain syndromes (17, 27).

### 1.2 Objectives

To investigate whether tDCS improves daily function and reduces pain levels in chronic headache sufferers.

## 2 Methods

### 2.1 Design and setting

This study was a two-arm, parallel-design, double-blind, sham-controlled RCT in which patients and assessors were blinded. A randomized, controlled trial was considered to be the most rigorous way of determining whether a cause-effect relationship existed between treatment and outcome measures (28). The study took place in the outpatient pain clinic at Vestfold Hospital Trust in southern Norway. Norwegians receive conventional medical treatment either highly subsidized or free of charge within the public health care system (29).

### 2.2 Sample

**Figure 1:**
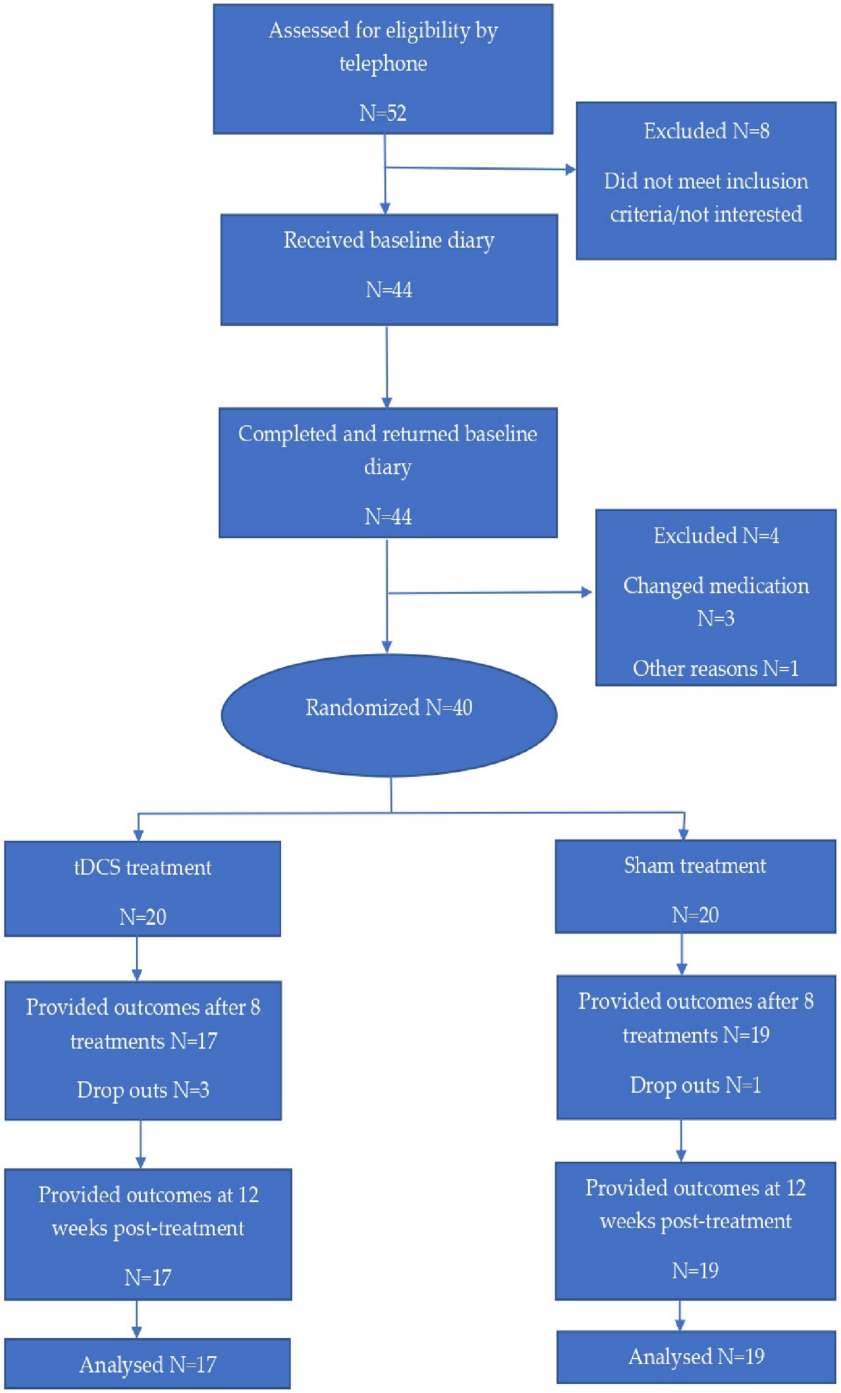
Flow diagram for subjects in the study.

Fifty-two patients, who were diagnosed with primary chronic headache according to the criteria of the International Headache Society (6), were referred by General Practitioners (GPs) and from the hospital's neurological outpatient department. Patients were recruited, included and followed up in the period August 2019–July 2021. Participant flow is demonstrated in Figure 1.

No differentiation between types of primary chronic headache was made. Patients not referred by a neurologist received a full clinical examination at the outpatient pain clinic and were referred for an fMRI scan of the head and neck. Baseline recordings regarding headache frequency were examined to confirm the diagnosis of primary chronic headache.

Before giving written consent, participants were informed in full of the aim and purpose of the study, as well as security measures concerning anonymity and publication. Further, that the study was voluntary and they were free to withdraw at any time.

The characteristics of the randomized participants are demonstrated in Table 1. Forty of the referred patients fulfilled the inclusion criteria of which 31 were female and 9 were male. All were Norwegian-speaking residents from southern Norway. Sixtyfive percent of the participants were employed (full-time 65%, part-time 35%). The remaining 35% received long-term sickness benefits or disablement benefits. Twentynine participants lived with a partner and the remaining 11 were single. Data concerning medication use and VAS scores was recorded for four consecutive weeks before starting treatment; there were no significant differences between the two treatment groups. MIDAS scores recorded before commencing treatment were comparable.

**Table 1:** Characteristics of randomized participants.

	tDCS *n* = 20	Sham *n* = 20
Age (mean ± STD)	48.6 ± 4.4	46.3 ± 6.1
Number of men	6	3
Number of women	14	17
No medication	3	4
One type of medication	Paracetamol (P) 500 mg Once a day *n* = 4 2–4 times a day *n* = 7	Paracetamol (P) 500 mg Once a day *n* = 5 2–4 times a day *n* = 5
Two types of medication	P + NSAID *n* = 3 P + antihypertensive *n* = 1 P + antidepressants *n* = 2	P + NSAID *n* = 4 P + antidepressants *n* = 2

### 2.3 Inclusion and exclusion criteria

Inclusion criteria: (I) participants between 18 and 70 years of age (II) diagnosis of chronic primary headache, defined by the International Headache Society 3rd Edition [IHS] (6), (III) recent neurological examination and fMRI scan of the brain and cervical spine (no longer than six months before inclusion), (IV) confirmed diagnosis of primary headache.

Exclusion criteria: (I) history of seizures, brain surgery, tumors, anomalies or deformities, (II) cardiac pacemakers, scalp metal implants, (III) severe psychiatric conditions, (IV) severe systemic disease, (V) pregnancy, (VI) current use of opioids, drug abuse, (VII) inadequate Norwegian language skills, (VIII) medication initiated less than three months before inclusion, and/or (IX) failure to complete the headache diary at baseline (less than 90% complete). (X) Diagnosis of chronic migraine.

Chronic headaches and migraine often overlap. Indeed, authors have long suggested that the variety of headaches in those with migraine might be a manifestation of the same underlying pathophysiology (30). Chronic migraine is defined as headache for more than 15 days per month lasting more than three months where at least eight of those headache days include at least two of the following symptoms: unilateral location, pulsating quality, and moderate to severe intensity. In addition, at least one of the following symptoms: nausea and/or vomiting, photophobia and phonophobia (7, 31). Since the diagnosis of most primary headache disorders continues to rely solely on clinical assessment, the diagnostic process can be complicated. There is a lack of specific diagnostic tests and biomarkers (8). Moreover, chronic headaches and migraine may coexist, further complicating the diagnostic process.

### 2.4 Sample size calculation

Estimates used in the sample size calculations were based on clinical experience. Pre-treatment: mean VAS = 8, SD = 1; post treatment: mean VAS = 4, SD = 2. Based on these results, with a difference of VAS 4 and SD = 2, only five participants were needed in each group (power: 80%, alpha = 0.05). We estimated that a very high degree of placebo contributed to these results. If the difference between the group results was less, i.e., from eight to five, total SD = 2, nine participants were needed in each group. To account for multiple measures and multiple tests, we lowered the significance level to 1%. Still keeping beta at 20%, 13 patients were needed in each group. To compensate for a possible 20% drop out, a minimum of 32 patients (16 in each group) were included.

### 2.5 Randomization and double blinding

The study secretary was responsible for the randomization allocation sequence and enrollment of participants who were randomized to the active tDCS and sham tDCS groups by using sequentially numbered, opaque sealed envelopes (28). Group allocation codes written on paper wrapped in aluminum foil were placed in sealed envelopes. The envelopes were then shuffled and placed in a black, non-transparent plastic bag. Study participants reached into the bag and delivered an envelope to the study therapist responsible for giving treatments (28). The study therapist was the only person in the study group with knowledge of group allocation; data regarding group allocation was locked up and verified (27). Interaction between the therapist and participants was kept to a minimum, as was contact between participants. Participants were blinded to treatment allocation (28). The tDCS device used in the trial included an automatic stimulation sham feature that produced a sham waveform based on the indicated “real” waveform so that the total run time matched the real case (17).

The principal researcher and the statistician were blinded to group allocation. Group allocation was revealed to the principal investigator only after all interim statistical analysis was completed in order to complete the final trial analysis (28).

### 2.6 Interventions

Direct current was applied through a pair of 35 cm^2^ rubber electrodes inserted into saline-soaked sponge pads using a Soterix Medical 1 × 1 Transcranial Direct Current Low-Intensity Stimulator apparatus [Model 1300A]. The anode was placed over the M1 and the cathode over the contralateral supraorbital area (17). M1 was identified by using the international 10–20 system of EEG electrode placement (31). The M1 on the left side of the head was routinely chosen for stimulation. Eight treatments of 30 min duration were carried out over a period of four consecutive weeks, with two or three days between each treatment for all patients. In the active treatment group, a direct current of two mA was applied for 30 min. The dose and frequency of stimulation were based on previous migraine research examining safety, comfort, and adverse effects (32–35).

The device included an automatic stimulation sham feature. The sham tDCS group received thirty seconds of stimulation at the start and end of the 30 min procedure. For the 29 min intervening, no stimulation was given (16).

### 2.7 Primary outcome measures

Data was collected at baseline on concluding eight treatments and again three months later. The primary outcome measure was change in function measured using The Migraine Disability Assessment Test [MIDAS] at the three time points (36), indicating the degree to which symptoms affect daily life. The MIDAS records lost days due to headaches in relation to employment, school, housework and family activities, social and leisure events. The questionnaire, based on five disability questions, was easily understood and quick to fill out. Total scores indicated four grades of disability ranging from none/little, mild, moderate, and severe. The MIDAS score was the sum of missed work or school days, household chores days, non-work activity days, and days where productivity was reduced by half or more (36).

#### 2.7.1 Secondary outcome measures

The predefined secondary outcomes were average pain intensity scores and changes in medication. All patients were asked to complete headache diaries for four weeks before randomization [baseline phase], during four weeks of treatment and during the following 12 weeks. Patients were supplied with a simple paper-sheet diary with columns to record the date, average pain severity during the corresponding 24 h period, and medication use. Average pain severity was estimated using a visual analog scale from 0 to 10, where 0 indicated no pain and 10—the worst possible pain. In addition, participants were asked to note any adverse effects, including a description of symptoms, the date of occurrence and duration of symptoms (on the reverse side of their diary sheet). This information was reported to the investigator at the following treatment session, or to the coordinator by phone during the follow-up period. Adverse effects were classified and graded according to the *Common Terminology Criteria for Adverse Effects* [CTCAE] (37).

### 2.8 Drop out

Patients were considered dropouts if they failed to undergo at least three consecutive sessions of tDCS or did not deliver study data post-treatment or at the 12-week follow-up point (38).

### 2.9 Statistical analysis

Statistical analyses were carried out using R [version 4.2.0] (39), based on the intention-to-treat principle (28). A linear mixed-effects model examined the differential treatment effects on MIDAS and VAS scores in the two groups. This model was considered appropriate for data analysis of repeated measurements and because of its capabilities related to missing data at certain time-points. This is particularly important considering that at least three out of four participants who dropped out did so due to adverse treatment effects. Omitting these subjects from the analysis could have potentially introduced bias. To address individual-level variability in baseline levels of MIDAS and VAS, we incorporated a random intercept in the mixed-effects model. This adjustment allowed individual-level variability and provided a more accurate representation of the data (39). Interaction terms [two groups × three time-points] were employed to examine the differences between groups in mean MIDAS and VAS scores between baseline [Pre] and post-treatment [Post], as well as between baseline and the three-month follow-up [three months]. Statistical significance was determined for differences with a *p*-value < 0.05.

## 3 Results

### 3.1 Primary outcome

**Figure 2:**
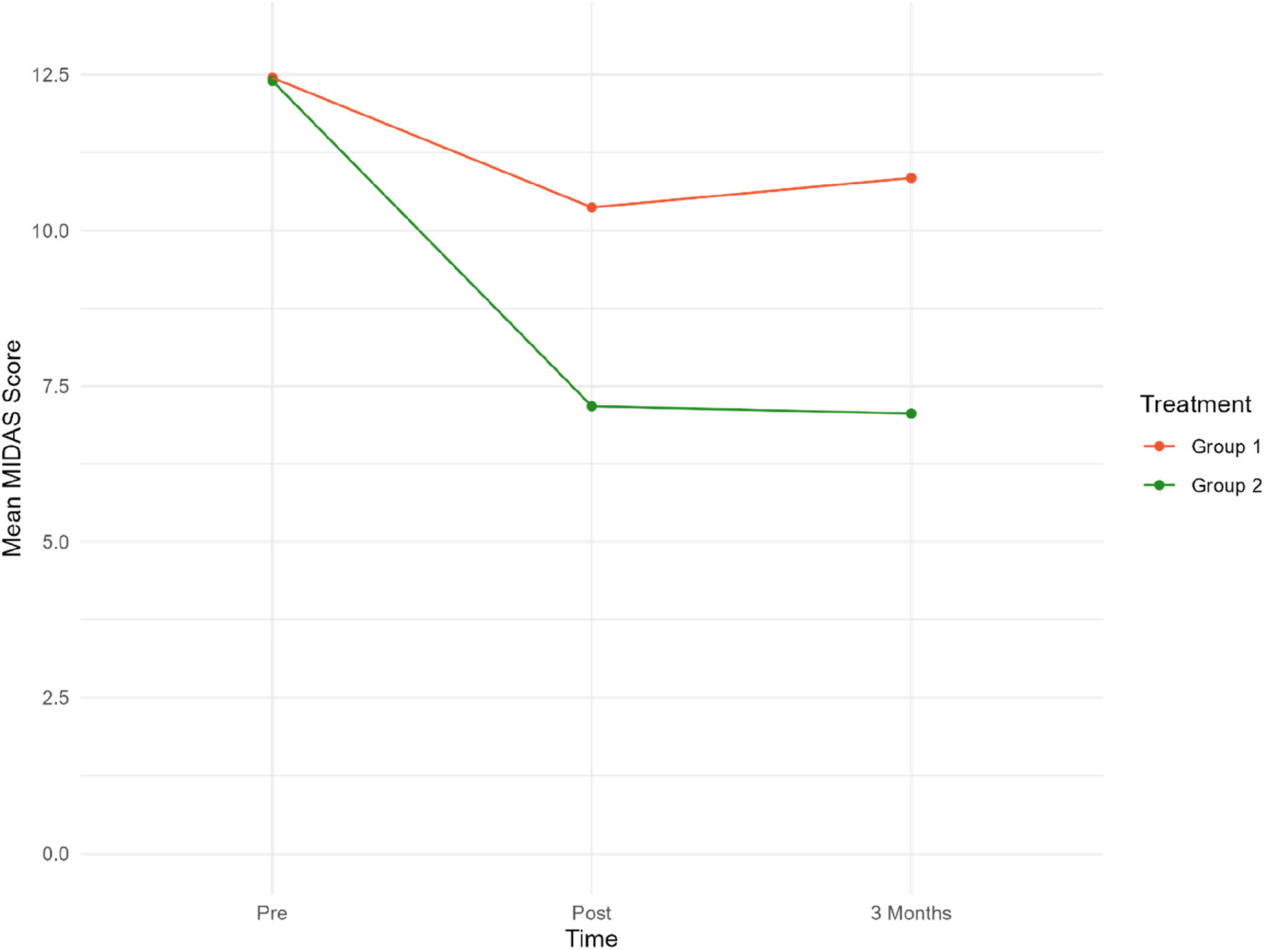
Mean MIDAS scores at pre/post-treatment and 3/three-month follow-up. Group 1 = Sham tDCS. Group 2 = Active tDCS.

Daily function levels are presented as mean MIDAS scores in Table 2; Figure 2. Table 2 presents the mean MIDAS scores, along with the corresponding standard deviation (SD) and number of participants (*N*) separately for the two groups at each time point.

**Table 2:** Mean MIDAS scores pre- and post-treatment.

Treatment	Time	Mean MIDAS	SD	*N*
Sham tDCS	Pre	12.45	5.38	20
Active tDCS	Pre	12.40	5.06	20
Sham tDCS	Post	10.37	4.75	19
Active tDCS	Post	7.18	5.57	17
Sham tDCS	3 months	10.84	5.51	19
Active tDCS	3 months	7.06	5.43	17

Mean MIDAS scores were reduced by 40% (*p *< 0.001) in the active tDCS group during treatment, from 12.40 at baseline to 7.18 post-treatment. A total reduction in mean MIDAS scores from pre-treatment to the three-month follow-up point of 41% (*p *< 0.001) was observed.

A significant reduction of approximately 14% from 12.45 to 10.47 (−1.76, *p* = 0.020) was observed in the sham tDCS group during treatment. However, there was no statistically significant decrease in MIDAS score between baseline and the three-month follow-up (12.45–10.84).

Overall, there was a statistically significant reduction in MIDAS scores in the active tDCS group compared to the sham tDCS group. Estimated MIDAS scores were significantly reduced during all measured time-periods in the active tDCS group, compared to sham tDCS. A reduction of 3.24 points (*p* = 0.004) was seen during the pre- to post-treatment period, and by 3.83 points (*p* = 0.001) from pre-treatment to three months after treatment (active tDCS v sham). These findings suggest that the treatment received by the active tDCS group significantly reduced MIDAS scores more effectively than the treatment received by the sham tDCS group.

Table 3. Presents MIDAS statistical test values. The table shows fitted values based on Linear mixed effects regression analysis.

**Table 3:** MIDAS statistical test values.

Time	Mean tDCS	Mean sham	Std. Error	DF	t-value	*p*-value	Mean difference	Lower	Upper
Pre	12.400	12.450	1.692	38	0.030	0.977	0.050	−3.282	3.382
Post	7.408	10.694	1.072	68	3.019	0.004	3.236	1.155	5.317
12 weeks	7.290	11.167	1.072	68	3.571	0.001	3.827	1.747	5.908

**Figure 3:**
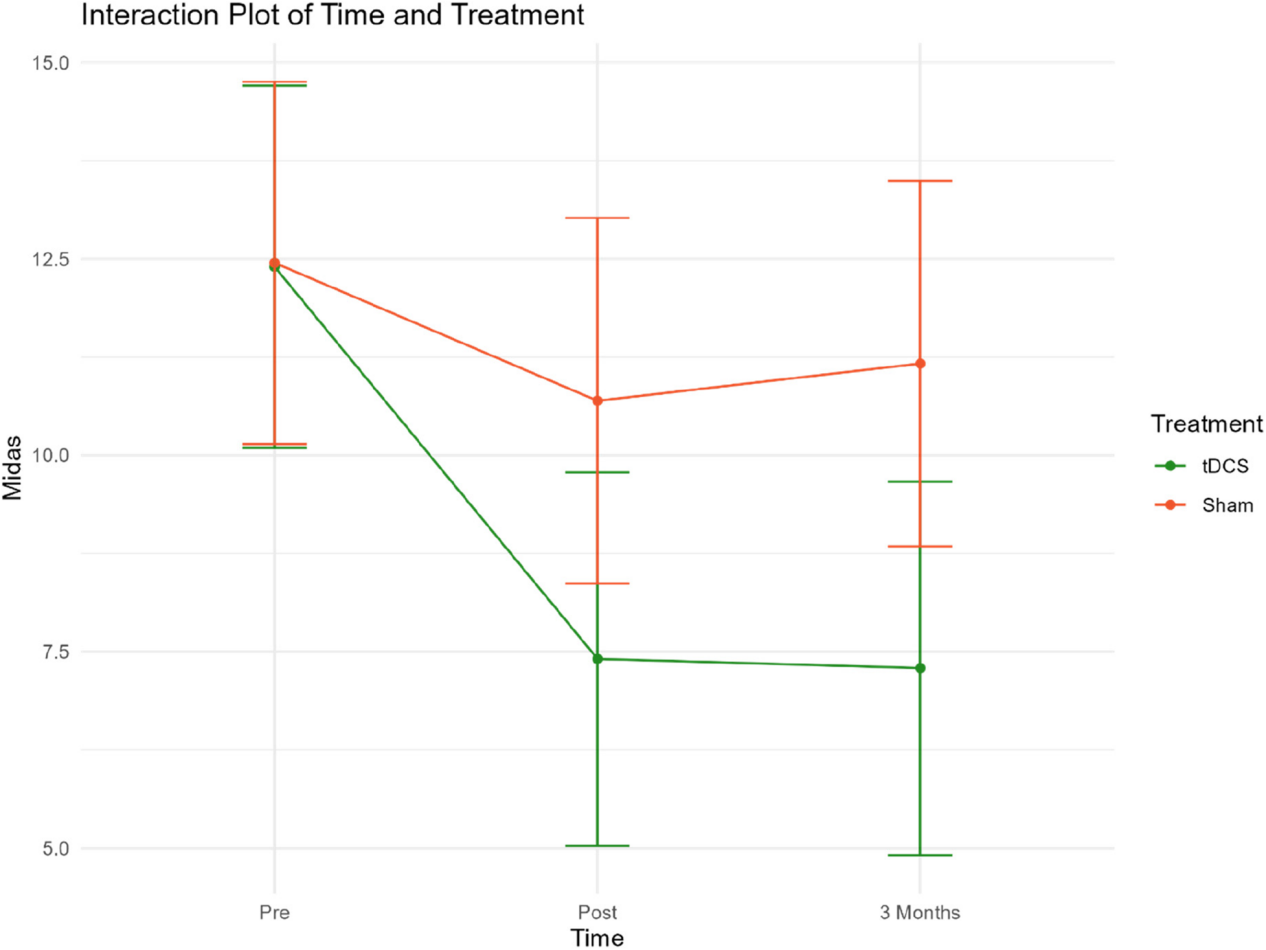
Interaction plot MIDAS.

An interaction plot for the two treatment groups related to treatment time points and MIDAS has been included in Extra material (Figure 3).

### 3.2 Secondary outcome measures

**Figure 4:**
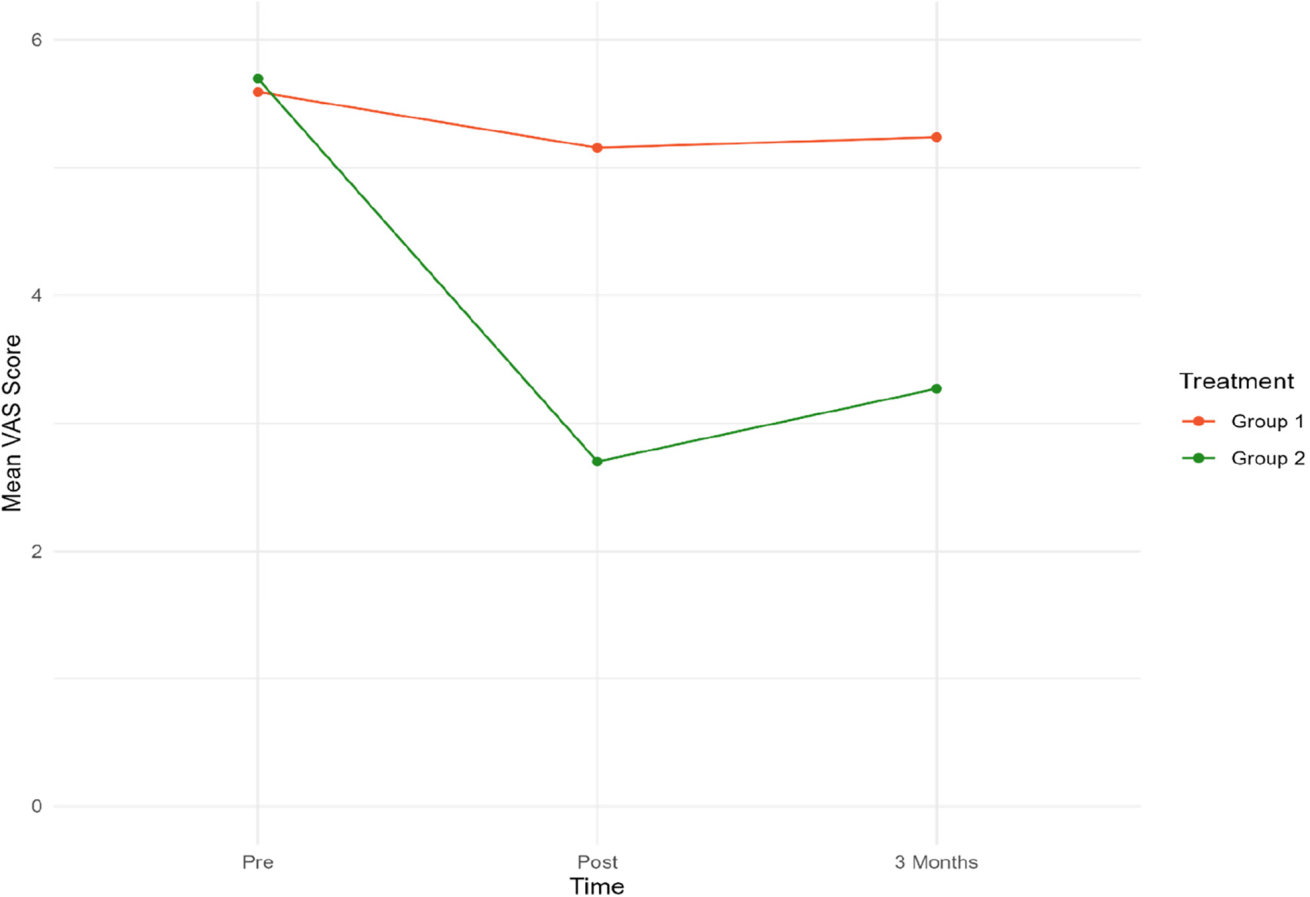
Mean VAS scores for both groups at pre-post and three-month follow-up. Group 1 = Sham tDCS. Group 2 = Active tDCS.

The secondary outcome measures were VAS scores and changes in medication. Figure 4 presents mean VAS scores at 3 time points. Table 4 presents mean VAS scores, along with the corresponding standard deviation (SD) and number of participants (*N*) separately for the two treatment groups, at each time point.

**Table 4:** Mean VAS scores pre-and post-treatment.

Treatment	Time	Mean VAS	SD	N
Sham tDCS	Pre	5.59	2.34	20
Active tDCS	Pre	5.70	1.50	20
Sham tDCS	Post	5.15	2.20	19
Active tDCS	Post	2.70	2.50	17
Sham tDCS	3 months	5.24	2.50	19
Active tDCS	3 months	3.27	2.67	17

At baseline, mean VAS scores were almost identical in both groups. A notable decrease in mean VAS scores from pre-treatment to both time points was observed in the active tDCS group. Specifically, VAS scores decreased by 55% (*p *< 0.001) from pre-treatment to post-treatment, and by 45% (*p *< 0.001) from baseline to three-month follow-up point. No significant reduction in VAS scores during treatment or the three-month follow-up period was observed in the sham tDCS group.

When the groups were compared, a statistically significant difference in favor of the active tDCS group was observed during treatment. On average, mean VAS scores were reduced by an additional 2.75 (*p* < 0.001) points in the active tDCS group from pre-treatment to post-treatment, and by an extra 2.27 (*p* < 0.001) points from pre-treatment to the endpoint three months later. No changes in medication were recorded in either group during treatment or follow-up.

Table 5. Presents VAS statistical test values. The table shows fitted values based on linear mixed effects regressions.

**Table 5:** VAS statistical test values.

Time	Mean tDCS	Mean sham	Std. Error	DF	t-value	*p*-value	Mean difference	Lower	Upper
Pre	5.695	5.590	0.718	38	−0.146	0.884	−0.105	−1.519	1.309
Post	2.561	5.211	0.515	68	5.352	<0.001	2.755	1.755	3.754
12 weeks	3.131	5.294	0.515	68	4.405	<0.001	2.267	1.268	3.267

**Figure 5:**
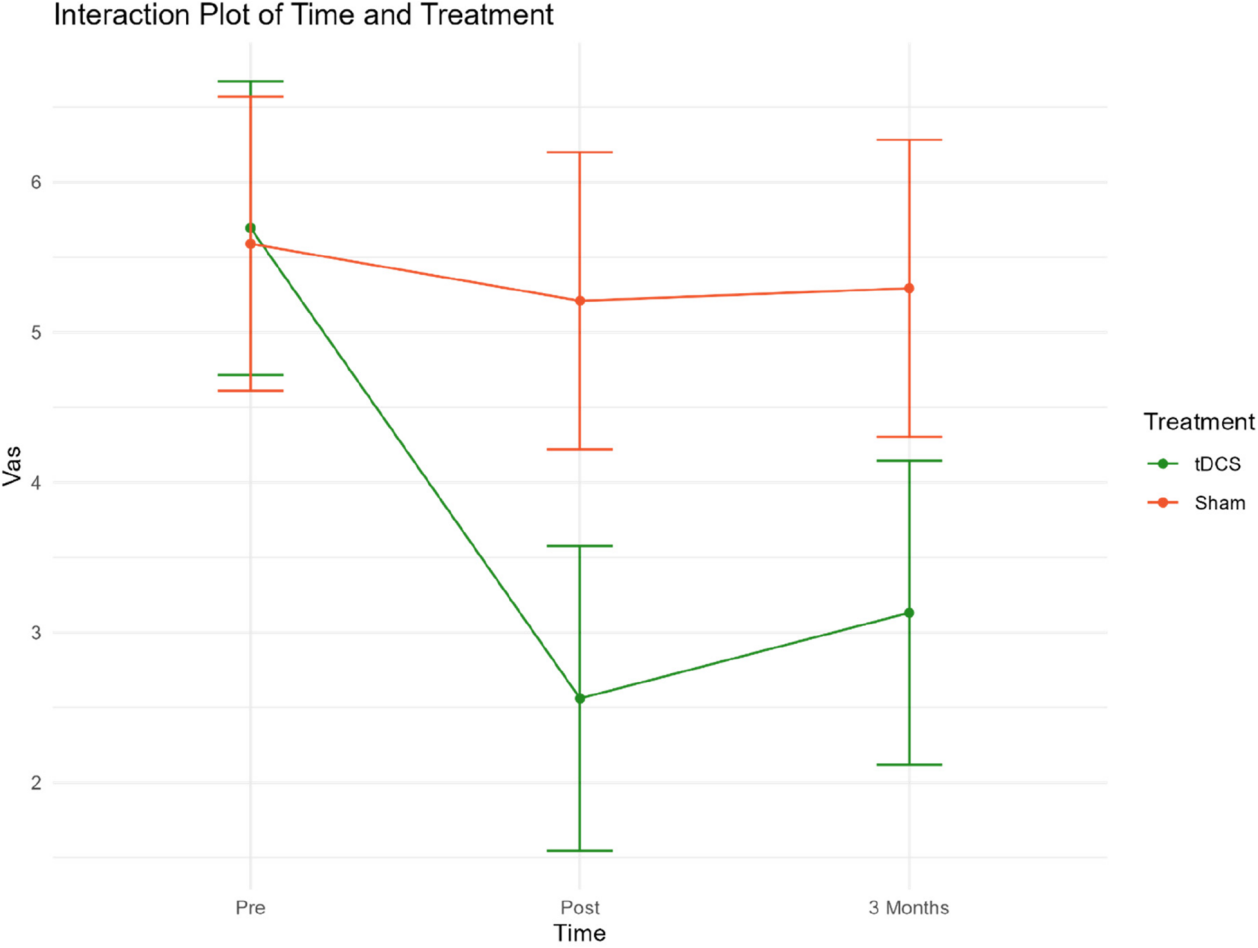
Interaction plot VAS.

An interaction plot for the two treatment groups related to time points and VAS has been included in Extra Material (Figure 5).

### 3.3 Relationship between MIDAS and VAS

VAS exhibited a significant association with MIDAS. Improvements in VAS were correlated with corresponding improvements in MIDAS scores. VAS did not demonstrate any significant interactions with time or the type of treatment, and three-way interactions were insignificant. These factors suggest that VAS had the same relationship with MIDAS irrespective of the type of treatment and time elapsed after treatment.

An additional noteworthy observation from Table 6 [extra material] is that any apparent relationship between MIDAS, time, and treatment type disappeared when controlling for VAS. This suggests that there is no direct significant relationship between active tDCS treatment, or sham tDCS and MIDAS score. A likely interpretation is that a reduction in VAS, representing a decrease in pain, served as the underlying mechanism through which the treatments influenced MIDAS. Improvements in MIDAS scores and reductions in pain levels measured at three-time points demonstrate the difference between treatments.

**Table 6:** VAS as a predictor of MIDAS.

	MIDAS SCORE			
Predictors	Estimates std	Error	*t-value*	*p*
Intercept	5.79	2.28	2.55	0.01 (3)
Time (post VSpre)	−0.36	1.57	−0.2 (3)	0.81 (9)
Time (3months VSpre)	−0.71	1.51	−0.4 (7)	0.64 (0)
Treatment (Active tDCS group)	−0.47	3.36	0.1 (4)	0.88 (9)
VAS score	1.19	0.36	3.33	0.00 (1)
Time (post treatment VSpre) × treatment active tDCS	−1.12	2.73	−0.4 (1)	0.68 (3)
Time (3 months VSpre) × Treatment active tDCS	−0.74	2.68	−0.2 (8)	0.78 (2)
Time (post treatment VSpre) × VAS score	−0.18	0.27	−0.6 (6)	0.50 (9)
Time (3 months VSpre) × VAS	−0.04	0.26	−0.1 (6)	0.87 (5)
Treatment (active tDCS) × VAS	0.05	0.53	0.10	0.92 (2)
Time (post VSpre) × treatment, active tDCS group × VAS	0.34	0.48	0.71	0.48 (3)
Time (3 months post tt VSpre) × treatment, (active tDCS group) × VAS	−0.09	0.46	−0.2 (0)	0.83 (8)

This table demonstrates linear mixed effects regression, with MIDAS score as outcome and VAS score as a predictor for MIDAS score predictors. Random Effects: *σ* = 2 3.05, *τ*00 subjects = 18.08, number of subjects 40, observations 112.

### 3.4 Medication use

No change in medication use was observed in either of the groups throughout four weeks of treatment and during the 12-week follow–up period. Most of the participants took Paracetamol 500 mg (*n* = 33). Twenty-one participants used only Paracetamol, 11 in the active tDCS group and 10 in the sham tDCS group. The remaining 12 Paracetamol users also took one of the following: a non-steroidal anti-inflammatory drug (*n* = 7 active tDCS, *n* = 3 sham tDCS), an antidepressant (*n* = 3 active tDCS, *n* = 1 sham tDCS 2), or an antihypertensive drug (*n* = 1 active tDCS). Seven patients did not use any medication.

### 3.5 Safety and adverse effects

Six participants reported adverse effects during the trial, of which five reported only one symptom. Two participants reported short-term dizziness [less than 30 min post-treatment], and two reported fatigue that lasted up to two days post-treatment. One person complained of a burning sensation at the anodal M1 application point. Lastly, one person reported short-term fatigue and a slight increase in headache intensity [less than 24 h post-treatment]. All symptoms were noted by the study therapist and graded as mild using the CTCAE 17 grading scale (37) by the principal investigator who was blinded to participant group allocation. Although the therapist observed redness on foreheads of all the participants on removal of the cathode***,*** none of the participants reported this symptom as an adverse effect. Since redness was observed in both groups, it was unlikely that it impeded the double blinding process.

Four participants withdrew from the study after randomization. Three of these were due to adverse effects, including tiredness [*n* = 1, sham tDCS], increased headache intensity [*n* = 1, active tDCS] and an itching sensation from the anodal electrode [*n* = 1, active tDCS]. The fourth person [*n* = 1, active tDCS] could not be contacted and thus the reason for discontinuation remains unknown. Three participants discontinued treatment after the second treatment session and the fourth discontinued treatment after the third session. The use of linear mixed effects models in the statistical analysis ensured the use of all available data at each time-point. Consequently, data from the four individuals who withdrew from the study was part of the model and the estimation of MIDAS and VAS at baseline.

Results of this study support the use of anodal tDCS to stimulate the M1. The procedure was well tolerated***,*** no serious adverse effects were recorded, hence making tDCS a safe non-pharmacological option. Similar findings have been reported in other studies investigating safety (45, 46).

#### 3.5.1 Timeline

Participants were included and followed up from August 2019 through to July 2021. No participants were included between February and October 2020, due to the Covid 19 pandemic.

## 4 Discussion

### 4.1 Participants, organization, and collaborations

This study investigated the effect of eight consecutive treatments of anodal tDCS over the M1 on daily function, pain levels, and the use of pain medication. Daily function was measured by recording the number of days where function was significantly reduced due to symptoms. The overall reduction in MIDAS score was 41% among participants receiving active tDCS, compared to 14% in the sham tDCS group. VAS scores were reduced by 45% from pre-treatment to the 12-week follow-up point. No changes in VAS scores were observed in the sham tDCS group. These improvements in daily function and headache pain levels suggest that tDCS is an effective treatment option for chronic headache sufferers.

Choosing changes in daily function as a primary outcome measure, was a direct result of subjective information the authors received when they carried out a qualitative study, where chronic headache sufferers were interviewed (40). Patients described a high level of disability, which led to emotional and behavioral changes, resulting in a serious reduction in daily activity and consequently quality of life. Similar findings were reported in a recent survey of 300 people suffering from non-cancer-related chronic pain conditions (41). The authors of this survey concluded that the extent to which pain interferes with daily life is the biggest threat to the mental health of people suffering from chronic pain.

Although The MIDAS was designed specifically for migraine sufferers, it was considered an appropriate tool to measure the degree to which chronic headache affects daily life (42). The MIDAS has demonstrated reliability in two separate population-based studies—in the United States and in the United Kingdom (43, 44). In addition to headache diaries, disability-graded information can be a useful tool regarding intervention choices in clinical practice, as well as increasing patient awareness regarding loss of function in their daily lives (43).

### 4.2 Previous tDCS studies

Research concerning tDCS as a treatment for headaches has focused on migraine. We were unable to find any studies focusing only on non-migrainous chronic headache. Neuromodulation, including tDCS, has been investigated in research studies over the past 25–30 years, and increasingly used clinically as a means of targeting CNS structures involved in neurological disease (47–50), depression (51), and pain syndromes (52–54). A recent systematic review and meta-analysis published evidence-based guidelines for the use of tDCS in neurological and psychiatric disorders (55). Based on tDCS efficacy and safety, recommendations were made for nine neurological and psychiatric disorders including migraine, neuropathic pain, fibromyalgia, and depression.

Another meta-analysis of eight studies [*n* = 153] investigating the effect of different non-invasive brain stimulation treatments for migraine reported a significant decrease in pain intensity, number of migraine attacks and painkiller intake after tDCS. Subgroup analysis for transcranial magnetic stimulation did not demonstrate similar results (56).

Clinical studies examining tDCS for migraine have shown conflicting results, despite being heterogeneous in terms of stimulation setup and patient clinical profile. Sample sizes are often small, the number of treatments varies, and follow-up period is often short. To find studies comparable to ours, we carried out a literature search to find RCTs with at least four weeks of treatment and at least 12 weeks of follow-up. Four RCTs were found where participants received four weeks of anodal tDCS at M1 and were followed up for between 12 and 16 weeks (32, 35, 57, 58). Sample sizes were small, *N* = 13, 37, 19 and 36 respectively. Outcomes were number of migraine attacks, pain intensity, and duration. All four trials reported a significant reduction in pain intensity lasting up to the follow-up points. Three of the trials (32, 35, 58) had 12-week follow-up points. DaSilva (33) reported a significant reduction in pain intensity at a 16-week follow-up point. No changes in medication were registered. Interestingly, none of the studies focused on daily function. However, results regarding pain intensity and medication use are comparable to our findings.

Studies investigating the safety of tDCS treatment for migraine (32–34, 56, 57) and chronic headache (34) have not revealed any safety issues, thus safety was not of concern in our study. The adverse effects reported in these studies are classified as mild, and typically include headache, rash, and drowsiness (35).

### 4.3 Implications for clinical practice and further research

This trial indicates that tDCS has the potential to reduce pain and improve daily function in chronic headache sufferers. tDCS provides a cheap, effective, easily tolerated treatment option which can be administered in an outpatient clinic. The development of home-use tDCS devices, which are relatively cheap, has the potential to provide individuals with an effective self-management strategy. Exploration of combined treatment strategies such as the use of tDCS and exercise, sleep hygiene, psychological interventions, or low-dose drugs, warrants attention. The encouraging results of this study merit confirmation in large-scale, randomized clinical trials.

### 4.4 Strengths and limitations

Significant improvements in daily function and pain levels and the occurrence of few, mild adverse effects contributed to an overall positive net effect in this trial. Treatments were quick, well tolerated and no sedation or monitoring during or after treatments was required.

Several limitations are present in this trial. Only 40 participants are included. Although the sample size is great enough to provide adequate statistical power, based on the sample size calculation, larger trials are needed to confirm our results. Secondly, although participants and assessors were blinded, the therapist carrying out treatments was not. This possibly introduced some degree of bias, regarding attitudes and differential treatment approaches. Thirdly, participants in the sham tDCS group received a total of 60 s of electrical stimulation and for the remainder of the treatment time [29 min] no stimulation was given. It is possible that this short 60 s stimulation time had some degree of effect, possibly reducing the difference between sham and active treatment group effects. As a control, this method is far from perfect (59). The issue of a sham intervention that is not inert is a common problem for several modalities, including acupuncture (60).

Research suggests that a significant placebo effect is associated with tDCS (61). A person's beliefs and expectations of a treatment are widely regarded as two of the strongest components of placebo effects (62). Furthermore, there is evidence that tDCS has the potential to increase the placebo effect by reinforcing brain networks activated by the expectation of benefiting from the treatment (63). Assessing psychological factors such as expectations of outcomes before inclusion may have provided more information regarding the influence of placebo on the two treatment groups in our study. However, the use of the same tDCS apparatus in both patient groups, which registered “real” and “sham” activity, with a visible electrode contact measurement scale, electrical stimulation light and timer; may have provided a degree of placebo equivalence.

tDCS is a passive treatment technique. Participants did not have the opportunity to contribute to any potential improvement and they did not receive any information regarding pain mechanisms. Our primary outcome measure was changes in daily function levels. It is widely accepted that exercise programs often lead to improved daily function and that psychological interventions can affect pain processing (54). Adding a third arm to this trial supplementing tDCS with either exercise and/or psychotherapy may have potentially provided participants with an increased feeling of involvement and control (64).

Participants in this trial were offered eight treatments over four weeks. An increased number of treatments may have improved results. However, statistically significant improvements in daily function and pain levels were recorded both during treatment and during the 12-week follow-up period. Covid 19 restrictions did not allow assessment of results beyond the 12-week follow-up.

No changes in medication were reported in either group, possibly due to the short follow up period. Participants experiencing an increase in function and reduced pain levels during the 12-week follow-up period may not have had the confidence to rely on further longer-term improvements. From a practical point of view, GP appointments for individuals wanting to discuss changes in their prescribed medication may have been difficult to access during the study period, as well.

Statistical analysis suggested that improvements in MIDAS were dependent on a decrease in VAS scores, indicating that a reduction in pain facilitated improvements in daily activity. Based on these findings, changes in pain levels may have been more suitable as a primary outcome measure.

## 5 Conclusion

Results from this trial suggest that tDCS has the potential to improve daily function and reduce pain levels in patients suffering from chronic headaches. Interventions were well tolerated, with no serious adverse effects. The International Association of Pain [IASP] and the National Institute of Health and Care Excellence [NICE] have recommended self-management techniques and non-pharmacological treatment approaches for sufferers of chronic primary pain. Results from this study indicate that tDCS has the potential to be a valuable part of an integrative self-care plan for chronic headache sufferers if the results of this trial can be confirmed in larger clinical trials.

## Data Availability

The raw data supporting the conclusions of this article will be made available by the authors, without undue reservation.
